# Conceptually Funded Usability Evaluation of an Application for Leveraging Descriptive Data Analysis Models for Cardiovascular Research

**DOI:** 10.3390/diagnostics14090917

**Published:** 2024-04-28

**Authors:** Oliver Lohaj, Ján Paralič, Zuzana Pella, Dominik Pella, Adam Pavlíček

**Affiliations:** 1 Department of Cybernetics and Artificial Intelligence, Faculty of Electrical Engineering and Informatics, Technical University of Košice, Letná 9, 040 01 Košice, Slovakia; jan.paralic@tuke.sk (J.P.); adam.pavlicek@student.tuke.sk (A.P.); 2 Center of Simulator and Virtual Medicine, Department of Medical Informatics and Simulator Medicine, Pavol Jozef Šafárik University in Košice, 040 11 Košice, Slovakia; zuzana.pella@upjs.sk; 3 1st Cardiology Clinic, Pavol Jozef Šafárik University in Košice, VÚSCH, Ondavská 8, 040 11 Košice, Slovakia; dominik.pella@gmail.com

**Keywords:** association rules, clustering, cardiovascular diseases, descriptive modeling, system usability scale, usability, Van Welie layered model of usability

## Abstract

The focus of this study, and the subject of this article, resides in the conceptually funded usability evaluation of an application of descriptive models to a specific dataset obtained from the East Slovak Institute of Heart and Vascular Diseases targeting cardiovascular patients. Delving into the current state-of-the-art practices, we examine the extent of cardiovascular diseases, descriptive data analysis models, and their practical applications. Most importantly, our inquiry focuses on exploration of usability, encompassing its application and evaluation methodologies, including Van Welie’s layered model of usability and its inherent advantages and limitations. The primary objective of our research was to conceptualize, develop, and validate the usability of an application tailored to supporting cardiologists’ research through descriptive modeling. Using the R programming language, we engineered a Shiny dashboard application named DESSFOCA (Decision Support System For Cardiologists) that is structured around three core functionalities: discovering association rules, applying clustering methods, and identifying association rules within predefined clusters. To assess the usability of DESSFOCA, we employed the System Usability Scale (SUS) and conducted a comprehensive evaluation. Additionally, we proposed an extension to Van Welie’s layered model of usability, incorporating several crucial aspects deemed essential. Subsequently, we rigorously evaluated the proposed extension within the DESSFOCA application with respect to the extended usability model, drawing insightful conclusions from our findings.

## 1. Introduction

Cardiovascular disease is a term affiliated with many health problems related to the heart and the vascular system. These medical conditions comprise an abnormal medical status that directly affects the heart and all its parts. From the available information, it can be concluded that cardiovascular diseases are the most common cause of death worldwide. Heart disease is the most significant health problem today. According to the World Health Organization (WHO) [1,2], solutions to cardiovascular diseases (CVDs) should be prioritized, as they have a global primacy in regard to mortality. According to the WHO, 17.9 million deaths from CVDs have been recorded, amounting to 32% of the total number of deaths. Within CVDs, 85% of heart attack and stroke-related deaths can be attributed to CVD-related heart conditions [1]. Based on statistics from the World Health Organization, up to 31% of global deaths are attributable to diseases and problems related to the heart and vascular system [1]. According to data from the European Heart Network [3], cardiovascular diseases are responsible for nearly 4 million deaths annually in Europe, with nearly two million of these occurring within the European Union alone. This means that CVDs account for 45% of all deaths in Europe and 37% within the European Union. Across most European nations, CVDs are the primary cause of death among men, and they are also the leading cause among women in nearly all countries. Geographically, Central and Eastern Europe experience higher mortality rates from coronary heart disease and stroke compared to Northern, Southern, and Western Europe. Despite this, there has been a gradual improvement in cardiovascular health across Europe, including in less economically developed regions, although this improvement contrasts with the alarming rise in CVD-related mortality at the turn of the 21st century. Annually, approximately 11.3 million new cases of cardiovascular disease are reported in Europe.

Based on data from 2023 State of Health in the EU—Slovakia [4], it is clear that, in Slovakia, the mortality from cardiovascular diseases is almost 100% higher than the OECD (Organization for Economic Co-operation and Development) average and 300% higher than in France, the Netherlands, and Italy. Notably, in Slovakia more than 36% of all deaths, meaning more than 26 thousand people, die annually from cardiovascular diseases, i.e., ischemic heart disease and sudden stroke, which represents approximately 40% of deaths in men and 50% of deaths in women. Slovakia has one of the highest mortality rates linked to preventable and treatable causes. There is still considerable room for improvement in terms of effective public health policies to reduce premature deaths. Although prevention and health promotion are part of the political agenda, funding is still insufficient.

When examining global data, it is important to focus on two distinct geographical regions: China and the United States of America. According to statistics from the World Health Organization, non-communicable diseases (those not transmitted by infection) account for 87% of adult deaths worldwide [5], with cardiovascular disease alone contributing to nearly half, or 45%, of these fatalities. Globally, China is the most affected country, with an estimated one in five adults in the country suffering from cardiovascular disease [6]. China stands out as a concerning leader in mortality due to these diseases. In the United States, a person dies from cardiovascular disease every 37 s [7], resulting in 647,000 deaths annually, representing one in four deaths. Unlike China, coronary artery disease ranks as the primary form of heart disease in the United States [8].

Viewed from a different vantage point, the gravity of the situation and the widespread prevalence of chronic diseases are readily apparent in the continuous influx of medical records and data each day. Leveraging data analytics, particularly through the application of descriptive models, presents a pathway to deciphering the factors influencing disease severity. Furthermore, it enables the identification of distinct patient cohorts based on their medical examination results. In this landscape, the development of an intuitive and user-friendly application tailored to supporting cardiologists’ research through descriptive modeling becomes paramount. The usability of the app is crucial in ensuring its effectiveness and widespread adoption. Additionally, integrating feedback mechanisms and continuous improvement processes can further refine the app’s usability over time, ensuring its relevance and effectiveness in the ever-evolving healthcare landscape.

## 2. Related Work

The use of data mining techniques in specific application areas, including medical environments, is largely varied. In some cases, predictive models dominate, while descriptive models dominate in others.

### 2.1. Use of Different Data Mining Techniques in Medicie

A relevant perspective on this issue is provided by the study [9] conducted by Md Saiful Islam et al., who shed light on the diverse applications of data mining in the medical and healthcare domain. By systematically analyzing published studies and literature spanning over a decade, the study reveals the prevalence of various approaches to data mining tasks during the period from 2005 to 2016. Their findings underscore the significant role of predictive modeling algorithms, which were employed in 43% of the scientific publications reviewed. These algorithms enable healthcare professionals to anticipate and predict outcomes, thereby aiding in early intervention and personalized treatment strategies.

Furthermore, the study highlights the emergence of prescriptive approaches, albeit to a smaller proportion (9%), suggesting a growing emphasis on not only predicting but also recommending optimal courses of action based on data insights. Descriptive modeling algorithms, which constituted 48% of the cases analyzed, play a crucial role in unraveling patterns, trends, and associations within healthcare datasets [10]. Among these algorithms, association rules and clustering algorithms emerged as being the most frequently used, underscoring their efficacy in identifying meaningful relationships and grouping similar patient profiles.

Delving deeper into the application areas, the study reveals a notable concentration of descriptive models in domains aimed at ensuring equitable access to healthcare across diverse population groups. These models not only facilitate the analysis and interpretation of disease patterns and risks but also provide decision support to healthcare professionals during critical junctures.

A particularly compelling example (cited in the study [9]) is the application area focused on analyzing and monitoring adverse events and reactions to medicines post-marketing. Here, descriptive models play an important role in swiftly identifying and responding to potential safety concerns, thereby safeguarding public health and enhancing pharmacovigilance efforts. Overall, the study underscores the multifaceted impact of data mining in healthcare, ranging from predictive modeling for prognoses to descriptive modeling for insight generation and decision support.

### 2.2. Use of Association Rules Concept in Medicine

This can be followed up with studies that directly confirm the claims made. The research team of Towfek et al. [11] implemented a methodology that incorporated medical ontologies and improved semantic measures to boost the accuracy and interpretability of the obtained association rules. Their method delivers clinically pertinent information into patient perspectives on mental health, the accessibility of mental health resources, and the influence of physical health on mental wellbeing by mapping medical notions to ontological items and examining semantic relationships. On the other hand, the research team of Patil et al. [12] focused on finding association rules, using the Apriori algorithm to identify significant factors that point towards diabetes indications over a five-year time horizon. It is also important to note that these were exclusively the records of patients no younger than 21 years of age. On the basis of the results, it can be argued that the factors largely influencing the indication of this disease include, in particular, high plasma glucose concentration, obesity, and the age of patients between 40 and 59 years.

The Apriori algorithm can also be used in conjunction with other chronic diseases, such as COVID-19. A wide range of symptoms have been identified in COVID-19, from mild colds to severe health complications that can lead to death. This issue was addressed by the research team of Meera Tandan et al. [13]. Thus, the main aim of the mentioned study was to identify patterns in symptoms in patients with COVID-19 disease divided into groups according to age, sex, presence of chronic diseases, and whether the patient had overcome or succumbed to the disease. The most significant rules formed by Apriori in terms of age groups and the symptoms of COVID-19 disease were defined as follows:

- Patients under 20 years of age = [conjunctivitis, severe rhinitis];
- Patients aged 20 to 45 years = [dry mouth, sore throat];
- Patients aged 45 to 65 years = [nausea, weakness];
- Patients aged 65 years and older = [anorexia, fever].

In patients over 45 years of age, heart-associated symptoms, i.e., heart failure, cardiac arrhythmia, myocardial infarction, and respiratory problems such as pneumonia or sore throat, accounted for the majority of the association rules. As we can see, many identified association rules include the most significant COVID-19 risk factors, which were also proven by practice during the pandemic. We can assume, that using association rules and Apriori algorithm is likely to yield an important result within our cardiovascular research as well.

### 2.3. Use of Clustering Concept in Medicine

Expanding further on the significance of healthcare research, authors of the article [14] tried to improve the performance of logistic regression and YOLOv4 algorithms, proposing the use of advanced parallel k-means pre-processing, which is a clustering technique that identified patterns and structures in the data. Their results demonstrated that the combination of advanced parallel k-means pre-processing and the neural engine processor resulted in a significant improvement in the performance of logistic regression and YOLOv4, making them more reliable for use in medical applications. Another study [15], conducted by Guo Q. et al., adds valuable insights into the nuanced understanding of hypertension and its management. By employing the k-means clustering algorithm, the research team aimed to delineate distinct groups of hypertensive patients based on a comprehensive dataset encompassing not only medical records but also lifestyle and behavioral attributes. The findings of their analysis underscore the heterogeneous nature of hypertension, shedding light on the diverse profiles and characteristics exhibited by patients suffering from this condition. Through the identification of unique clusters, the study illuminates the variability in disease presentation and risk factors among hypertensive individuals.

One noteworthy revelation from the clustering analysis is the delineation of specific patient cohorts with distinct demographic and clinical profiles. For instance, the largest cluster, consisting of 172 patients comprising predominantly young male smokers, exhibits concerning cardiovascular risk factors, including elevated blood pressure levels and low HDL cholesterol, despite the absence of diagnosed coronary artery disease. This subgroup warrants targeted interventions aimed at smoking cessation and blood pressure management to mitigate their heightened cardiovascular risk. Conversely, the smallest cluster, composed of 70 mainly older women, presents a contrasting clinical profile characterized by lower diastolic blood pressure and elevated glucose and cholesterol levels. This subgroup may necessitate interventions focusing on glycemic control and lipid management to mitigate their cardiovascular risk and improve long-term outcomes.

By elucidating the heterogeneity within hypertensive populations, the study underscores the importance of personalized medicine in the management of chronic cardiovascular diseases like hypertension. With the aid of knowledge of distinct patient clusters, healthcare providers can tailor treatment strategies and interventions to address the specific needs and risk profiles of individual patients, thereby optimizing clinical outcomes and enhancing patient satisfaction. Overall, based on the research conducted by Guo Q. et al. [15], we can conclude that using descriptive models in cardiovascular research and management can yield important information and results that can be applied in practice to help save many lives.

### 2.4. Defining Usability

Usability was first used as a term more than 40 years ago when it replaced the term user-friendly [16]. In another study [17], authors talk about the dominant perspective that defines usability as quality of use. The quality of use perspective hypothesizes that the usability of a product varies depending on who is using the product, how they are using it, and for what purpose it is being used. The view of interaction with a product that needs to be considered when specifying usability is known as the context of use [18]. According to the International Organization for Standardization (ISO) and the norm ISO 9241-11:2018 [19], usability is defined as “*the extent to which a product can be used by specified users to achieve specified goals with effectiveness, efficiency and satisfaction in a specified context of use*”.

If we look deeper into usability, it encompasses the effectiveness, efficiency, and satisfaction with which users can interact with a product, system, app, or service. It extends beyond mere functionality to include the overall user experience, focusing on factors such as accessibility, learnability, and user satisfaction. A usable design prioritizes the needs and preferences of its users, striving to minimize cognitive load, streamline interactions, and facilitate intuitive navigation [20]. Usability testing and evaluation methodologies are crucial to identifying and addressing potential usability issues, as well as ensuring that the product or system meets the functional requirements and enhances user productivity and satisfaction. Ultimately, usability plays a vital role in determining the success and adoption of a product or service, as it directly impacts user engagement, trust, and loyalty. In fields such as healthcare, where usability can significantly affect patient safety and outcomes, a strong emphasis on usability is essential to ensuring effective and efficient delivery of care. Thus, usability serves as a main point of user-centered design principles.

To sum up the definition of usability and how we can use and implement it, in the realm of user-centered design, usability stands as a cornerstone principle. It encompasses the entire spectrum of a user’s interaction with a product, system, or service. A product with high usability empowers users to accomplish their desired tasks effectively, efficiently, and with satisfaction. This translates to clear interfaces, intuitive navigation, and a design that minimizes errors and learning curves. Ultimately, strong usability fosters positive user experiences, which are critical for product adoption and long-term success. Therefore, if we want to create a high-usability product, we need to implement all important aspects of usability during the development of an app, test it rigorously, and then implement changes based on feedback received during the testing phase. This will ensure that the app we develop is going to be used and that end-users will be satisfied with it.

### 2.5. Defining Usability of Decision Support Systems

The usability of decision support systems (DSSs) is crucial for ensuring their effectiveness and acceptance among users in various domains, ranging from healthcare to finance and beyond. These systems are designed to assist users in making informed decisions by analyzing complex data and providing relevant insights and recommendations [21]. A key aspect of usability in DSSs involves the accessibility, comprehensibility, and interpretability [22] of the information presented. Users need to easily navigate through the system, understand the data being analyzed, and interpret the recommendations provided.

A user-friendly interface with intuitive navigation and clear visualizations can greatly enhance usability, enabling users to interact with the system seamlessly and make decisions confidently. Furthermore, the efficiency of decision-making processes is heavily influenced by the usability of DSSs [23]. Users should be able to access the information they need quickly and efficiently without being bogged down by unnecessary complexity or cumbersome interfaces. Streamlined workflows and customizable features can improve efficiency by allowing users to tailor the system to their specific needs and preferences. Additionally, usability encompasses the responsiveness and reliability of the system, ensuring that users can rely on it to deliver accurate and timely insights when needed most.

Usability testing plays a critical role in optimizing DSS usability by identifying usability issues and gathering feedback from users. Through iterative testing and refinement, designers can fine-tune the system, and, moreover, ongoing usability evaluation and monitoring are essential for ensuring that the system continues to evolve in response to changing user needs and technological advancements [24]. Usability testing plays a crucial role in the development and refinement of DSSs, ensuring that these systems effectively meet the needs of users and facilitate informed decision making. Through usability testing, designers can gather invaluable insights into how users interact with a DSS interface, identify usability issues, and iteratively improve the system’s design.

One key aspect of usability testing in DSSs involves assessing the ease of use and the effectiveness of the system’s features and functionalities [25]. This includes evaluating the clarity of information presentation, the intuitiveness of navigation, and the efficiency of task completion. By observing users as they perform tasks within a DSS, designers can identify areas where the interface may be confusing or cumbersome, allowing them to adjust and improve usability. Usability testing also provides an opportunity to gather feedback directly from users regarding their experience with the DSS. This feedback can offer valuable insights into user preferences, pain points, and areas for improvement. By incorporating user feedback into the design process, designers can ensure that a DSS aligns with user needs and preferences, ultimately enhancing user satisfaction and adoption. Moreover, usability testing allows designers to assess the accessibility of the DSS interface, ensuring that it is usable by individuals with diverse abilities and needs [26]. This may involve testing the system with users who have different levels of technical proficiency or who rely on assistive technologies, such as screen readers or alternative input devices. By designing for accessibility, designers can ensure that a DSS is inclusive and can be effectively used by all users.

By iteratively testing and refining the DSS interface based on user feedback [27], designers can ultimately create systems that empower users to make better decisions and achieve improved outcomes. Usability is paramount in the design and implementation of decision support systems. By prioritizing accessibility, efficiency, and reliability, designers can create DSSs that empower users (in our case, cardiologists) to make better decisions, ultimately leading to improved outcomes and organizational success.

Usability can be measured in many ways, such as with SUS, MUSiC, or TeSS. SUS, or System Usability Scale [28], is a widely used questionnaire for assessing the perceived usability of a system. It consists of 10 questions designed to evaluate the user’s subjective perception of various aspects of usability, such as ease of use, learnability, and efficiency. Users rate each question on a 5-point Likert scale, and the scores are then converted and aggregated to produce an overall usability score ranging from 0 to 100. SUS provides a standardized method for quantitatively measuring usability and is commonly used in user experience research and usability testing. We define and explore SUS more in depth in chapter V.B.

The objective of the MUSiC project [29], or Metrics for Usability Standards in Computing, has been to develop a set of metrics-based methods that can be used individually and together to both specify formal requirements for the usability of a product and to assess whether a product meets those requirements. The project has defined usability in terms of the quality of use of a product, and it has developed tools and procedures for measuring usability.

In the context of usability, “TeSS” [30] refers to the “Tool for Exploring the Structure of Standards”, which is used for analyzing and visualizing complex standards or ontologies. This tool aids in understanding the structure and relationships within standards, making them more accessible and usable for stakeholders.

### 2.6. Van Welie Layered Model of Usability

**Figure 1:**
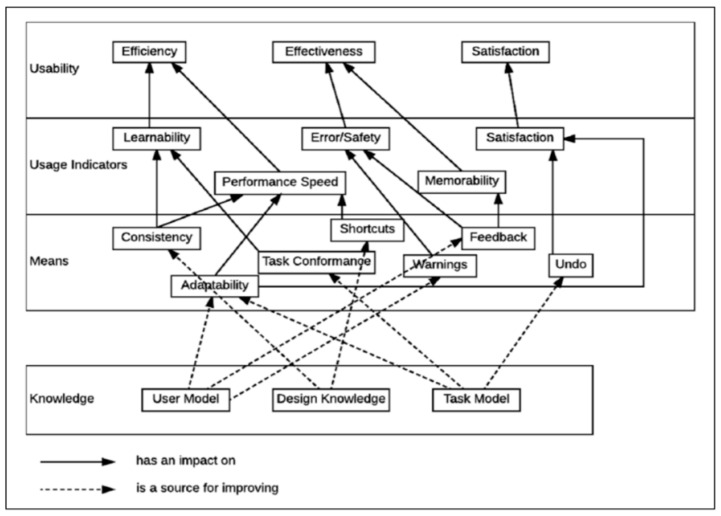
Van Welie layered model of usability (from [31]).

For the purpose of evaluating the usability of clinical decision support systems (CDSS), the Van Welie usability model according to the study [31] was used, which divides three aspects of usability into three layers, as seen in Figure 1. The first of them (counting from the top of Figure 1) includes the main three aspects of usability, e.g., effectiveness, efficiency, and satisfaction. The second layer specifies specifically Usage Indicators which are assigned to individual aspects from the first layer. These include indicators such as learnability, memorability, safety/errors, speed of performance, and satisfaction. Sheiderman [32] named this layer as “*the five measurable human factors aimed at evaluating goals*”. The third layer provides Means by which the indicators can be measured. These include, for example, consistency, availability of rollback operations, warnings, presence of feedback, and adaptability. This layer is specified by factors [31] that influence the concept to which they belong. For example, consistency positively affects learnability, and warnings can reduce mistakes/errors. It is important to study the consensus regarding usability, as this is one of the prerequisites for the success of using the DSS. This success depends on the design, functionalities, and features of the system. It addresses the needs of users and the behavior of the system that are part of the software. Improving usability is key, as poor usability is a major obstacle to successful system integration.

Hardenbol et al. in [33] studied 24 papers that covered at least one aspect of usability in CDSS related to medication in an outpatient setting. The findings showed positive efficacy results in 90 percent of the studies. The aim of the authors of the research was to summarize the results within three aspects of usability and to examine current usability evidence based on Van Welie indicators and means of use in CDSS evaluation research. These indicators were later analyzed to determine which usability categories in the ISO standard require further investigation.

To investigate the relationships between the three aspects of usability, Van Welie’s indicators, and means of use, the authors synthesized each study according to his model in each category. The results of this research showed that some indicators of the use of the Van Welie model, such as memorability and learnability, have scarcely been studied, but also they are essential components of effectiveness and efficiency and should therefore be included and developed in future research on usability [33]. A study that looked at examining usability in software [18] points out that learning ability can be measured by comparing the quality of use for users over time or by comparing the usability of a product for experienced and inexperienced users. It follows from the article [33] that Means for measuring Usage Indicators (Figure 1) and, consequently, usability measurements vary considerably between studies, and also that most studies have focused on examining warnings rather than means of feedback.

A possible reason for this may be that providing feedback is a task that requires more knowledge of the situation and, therefore, the generation of alerts is easier to implement. It is believed that generating feedback can lead to improved CDSS because it can improve doctors’ decision-making processes instead of just warning them when they perform something incorrectly. Evidence of usability of the medication-related CDSS shall be based on the study by Hardenbol et al. [33], which talks about usability aspects of medication-related decision support systems in the outpatient setting, as these could be found mainly for efficacy and showed a high percentage of positive results in reducing medication errors, which is consistent with previous studies. However, the results in the categories regarding efficiency and satisfaction diverged too much to draw conclusions, and the two categories remain insufficiently studied.

After reviewing several studies focused on usability research, its aspects, and specific metrics, we concluded that there is no universal model by which we can measure usability. As usability is a very complex concept, and we need to consider in which environment, system, and with which users we need to evaluate it, it is necessary to choose appropriate metrics and methods to measure usability. Therefore, in this part of research, we summarized current knowledge before, in the next part, proposing a new, more universal usability measurement model for a data analytics-based decision support system, according to which we can more easily determine which methods are suitable for usability measurement based on our requirements. We will start from Van Welie’s model, which is still generally in use but lacks many new aspects of usability that have become important in the last few years. We will extend the model with specific indicators of usability aspects that have their own measurement methods.

## 3. Characteristics of Descriptive Models

In the previous section, we mentioned a few terms that should be briefly explained.

### 3.1. Descriptive Modeling

The descriptive approach, often referred to as descriptive modeling, constitutes a fundamental methodology in data analysis that aims to illuminate underlying patterns and structures within a dataset [34]. Unlike predictive models, which predict future outcomes based on historical data [35], descriptive models focus on elucidating the inherent characteristics and trends present in the input data themselves [34]. By employing techniques such as clustering, association rules, and summarization, descriptive analytics provides valuable insights into the nature of the data, enabling stakeholders to discern meaningful patterns and relationships.

One distinguishing feature of descriptive analytics is its emphasis on the exploration and understanding of data without necessarily predicting future outcomes. While predictive models rely on defining a specific target attribute to forecast future events [35], descriptive analytics is not constrained by such a requirement. Instead, it allows analysts to delve into the nuances of the dataset, uncovering hidden insights and informing decision-making processes. Moreover, descriptive modeling plays a pivotal role in various domains [34], including business intelligence, healthcare, and finance, where understanding past and present trends is essential for strategic planning and decision support. In essence, the descriptive approach serves as a foundational pillar in the world of data analytics, offering a comprehensive framework for exploring and interpreting data patterns, thereby facilitating informed decision making and driving organizational success. Association rules and clustering are among the most often used descriptive models.

### 3.2. Association Rules

Association rules serve as a powerful technique in data mining and analysis, primarily focusing on uncovering frequent and meaningful relationships between individual attributes within a dataset. By examining patterns of co-occurrence among different variables, association rules provide valuable insights into the underlying associations and dependencies present in the data [36]. This enables analysts to gain a deeper understanding of the complex interactions between variables, shedding light on hidden patterns and trends that may not be immediately apparent. One of the key advantages of association rules lies in their simplicity and ease of interpretation. Unlike some other data mining techniques that may produce complex models or require advanced statistical knowledge to interpret, association rules offer straightforward and intuitive insights into the relationships between variables, which is also important for our research.

If the insights are intuitive and easy to understand, end-users will find this app usable. This simplicity also enables stakeholders from various backgrounds to grasp the implications of the discovered associations quickly, making it a valuable tool for decision making and problem solving in diverse domains. Association rules provide a flexible framework for uncovering meaningful connections between variables. This adaptability makes association rules a valuable asset for organizations seeking to both extract actionable insights from their data and drive informed decision making.

### 3.3. Clustering

Clustering can be characterized as a method for finding relationships and connections between objects in a dataset before then classifying them into groups based on similar properties; these groups are also called clusters. Objects within one cluster display many more similarities to each other than to objects from different clusters [36]. Clustering is a cornerstone method in data analysis that is known for its capacity to reveal intrinsic relationships and structures within datasets. By utilizing clustering algorithms like k-means, hierarchical clustering, and density-based clustering, analysts can partition data into cohesive clusters based on similarities among data points. This enables the identification of patterns and associations, offering invaluable insights into the underlying structures of the data. From market segmentation in marketing to document clustering in natural language processing, clustering algorithms provide a flexible framework for organizing and understanding complex datasets. This adaptability makes clustering a go-to tool for exploratory data analysis, pattern recognition, and decision support across various industries. Additionally, clustering serves as an effective means of data reduction and summarization, condensing large datasets into manageable clusters. This facilitates streamlined data interpretation and visualization, empowering stakeholders to extract actionable insights and make informed decisions based on revealed patterns.

#### 3.3.1. Hierarchical Clustering

Hierarchical clustering algorithms [36] represent a fundamental approach in data analysis, offering a hierarchical partitioning of objects within a dataset. This hierarchical structure allows for a nuanced understanding of the relationships and similarities between data points, offering valuable insights into the underlying structures of the data. These algorithms can be broadly categorized into two main groups: agglomerative and divisive.

Agglomerative hierarchical clustering [37] follows a bottom-up approach, starting with each data point as its own cluster and iteratively merging clusters based on their similarities. At each step, the algorithm identifies the two closest clusters and combines them into a single cluster, gradually forming a hierarchy of nested clusters. This process continues until all data points are grouped into a single cluster, resulting in a dendrogram that illustrates the hierarchical relationships between clusters. Conversely, divisive hierarchical clustering [38] employs a top-down approach, beginning with all data points grouped into a single cluster and recursively splitting clusters into smaller clusters based on their dissimilarity.

The choice between agglomerative and divisive hierarchical clustering depends on the nature of the dataset and the specific objectives of the analysis. Agglomerative clustering is often preferred for its simplicity and efficiency, particularly when dealing with large datasets, while divisive clustering may offer greater interpretability and control over the clustering process. Hierarchical clustering algorithms provide a powerful framework [38] for organizing and understanding complex datasets, offering insights into the hierarchical relationships between data points.

#### 3.3.2. Clustering Based on the Selection of Cluster Representatives

Clustering algorithms that operate based on the selection of cluster representatives employ a fundamental principle where cluster centroids are initially formed randomly before individual elements are then iteratively moved within or between clusters to the nearest centroid to refine the clustering arrangement. The criterion for determining whether an element belongs to a particular cluster typically relies on its distance from the center or representative of the cluster. This distance metric is often calculated using measures, such as Euclidean distance or cosine similarity, that depend on the nature of the data and the clustering algorithm being utilized [39]. Central to this approach is the concept of the centroid, or center of gravity, which represents the average position of all data points within a cluster. The centroid serves as a reference point for assessing the similarities or dissimilarities of individual data points to the cluster they belong to.

Prominent clustering algorithms that operate based on this principle include k-means and Partitioning Around Medoids (PAM), also known as k-Medoids [40]. In k-means clustering, the centroids are iteratively updated to minimize the total intra-cluster variance, resulting in clusters with tight boundaries and well-defined centroids. PAM, on the other hand, selects representative data points, known as medoids, as the representatives of clusters, making it more robust to outliers and noise in the data. Clustering methods based on the selection of cluster representatives offer a flexible and scalable approach to partitioning data into coherent groups. By iteratively refining cluster assignments based on proximity to centroids or medoids, these algorithms enable the identification of meaningful patterns and structures within datasets, facilitating data exploration, analysis, and interpretation across various domains and applications.

## 4. Design, Development, and Characteristics of the Implemented System

### 4.1. The Idea of the Implemented System

There are many examples of applications of descriptive models. Drawing from the preceding analysis, it is evident that descriptive data mining, or descriptive models, hold significant relevance across diverse sectors within the medical and healthcare domains. Our goal and motivation were to design and develop a comprehensive system where the user would be able to search and analyze association rules while also applying clustering methods to the input dataset (either one of their choice or the one built-in) and extracting relevant information from the obtained results. The final version of the implemented system solution corresponds to this idea and can be divided into the following parts:

- Data set retrieval—although it is stated in the introduction of this article that the descriptive models implemented in the application are primarily applied to a specific group of patients from the East Slovak Institute of Heart and Vascular Diseases, we decided to add a functionality that also allows the user to work with their own dataset.
- Search for association rules—this part of the application is designed exclusively for searching and subsequent analysis of association rules, and we used the Apriori algorithm for this task.
- Applying clustering methods—within this section a user can select one of the offered clustering methods, with the choices including hierarchical clustering or clustering using PAM and k-means algorithms, and apply it to the input dataset, giving the user many outputs for obtaining valuable information.
- Search for association rules within defined clusters—the last part of the application directly follows the previous one and is based on it, whereby the user has the possibility, again using the Apriori algorithm, to search for and subsequently analyze association rules within individual clusters that have been identified by applying the chosen clustering method.

In this part of research we will focus on the presentation of key information from the design and development phases, the functionalities of the implemented software, and the characteristics of the individual parts of the application. It is also important to note that we have used an evolutionary model of the development life cycle in the design and subsequent implementation of the software solution.

### 4.2. Development Environment

In the implementation of the application, the choice of the R programming language proved to be highly advantageous. R is an open-source language that offers a comprehensive suite of tools for data processing, analysis, and visualization. Its extensive collection of packages and libraries makes it particularly well-suited for a wide range of tasks, from basic data manipulation to advanced statistical modeling and machine learning. One of the key strengths of R is its suitability for the development of interactive dashboards and applications. With packages such as Shiny, R users can easily create dynamic and interactive web-based interfaces that allow for real-time exploration and visualization of data. This capability proved invaluable in our application, as it enabled us to develop user-friendly interfaces that facilitated intuitive interaction with the underlying data and algorithms.

Throughout the implementation process, we leveraged a variety of R packages to streamline different aspects of the software development pipeline. For data processing and manipulation, packages such as *dplyr* and *tidyr* provided powerful tools for data wrangling and transformation. For visualization of results, ggplot2 emerged as a go-to choice, offering elegant and customizable plots that effectively communicated complex findings. Additionally, for the application of clustering algorithms, packages such as *cluster* and *factoextra* offered efficient implementations and convenient interfaces for model fitting and evaluation. Use of the R programming language, coupled with its extensive ecosystem of packages, significantly facilitated the development and deployment of our application, which we named DESSFOCA.

### 4.3. Application User Interface

**Figure 2:**
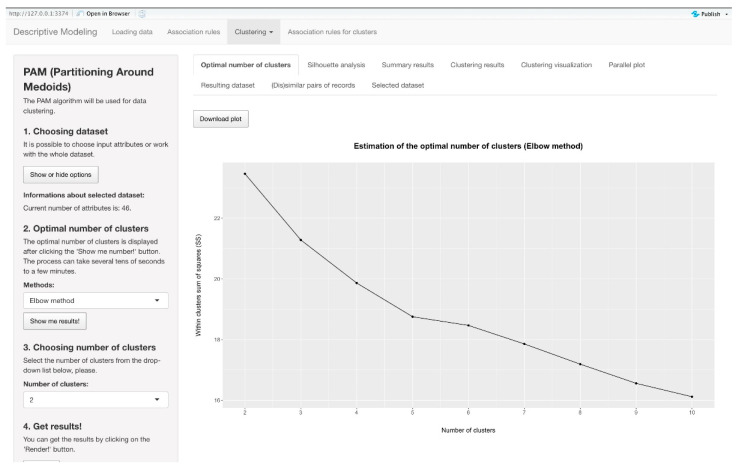
Screenshot from the developed app showing Clustering tab.

In the implementation of our app, the utilization of the aforementioned R Shiny package proved instrumental, particularly in regard to its aptitude for crafting web applications. The user interface design was structured into three primary sections (see Figure 2), each serving distinct purposes. Firstly, the menu positioned at the top of the screen provided easy navigation and access to various core functionalities of the app. This menu served as a centralized hub for users to initiate actions and explore different features of the application. The menu also contained four main navigation buttons: *Loading data*, *Association rules*, *Clustering*, and *Association rules for clusters*. These buttons stayed on the screen throughout the duration of use and did not change, so the user could switch to a different data analytic method or decide to load a new data set and start over at any given time.

Adjacent to the main content area, on the left side of the screen, resided the sidebar. This sidebar served as a convenient space for housing supplementary tools, options, and controls. Its strategic placement ensured quick access to frequently used functions, enhancing user efficiency and workflow. Users could easily toggle between different sections of the application or customize settings from the sidebar, which contributed to a seamless and intuitive user experience. This sidebar on the left side of the screen was present at any given time, dynamically changing with the activity or part of the app the user was working on within that moment while always containing important settings for the given part of the app. There was also a button, “*Show or hide options*”, that we implemented so that users could view more options if they needed to try different settings (or toggle them when they were not needed anymore). We also implemented a self-explanatory numbered system in the side bar so that the users could logically proceed in settings.

The main section, occupying the rest of the screen, served as the focal point of user interaction. This expansive area accommodated the core functionalities and content of the application, providing ample space for data visualization, analysis, and exploration. Within this main section, specific components, such as the data retrieval section, were meticulously designed to facilitate user interaction and data manipulation.

For instance, the data retrieval section was the only one different from all other sections, as it featured a menu that allowed users to specify their data retrieval criteria, such as selecting datasets or defining query parameters. The main section of this data retrieval component housed various interactive elements, such as input fields, dropdown menus, and action buttons, enabling users to customize their data retrieval process according to their specific needs and preferences. This modular design approach ensured that users could efficiently navigate through the data retrieval process while making informed decisions and extracting valuable insights from the available datasets. The structured layout of the user interface (comprising the menu, sidebar, and main section, as well as specific components like the data retrieval section) played a pivotal role in enhancing user engagement, facilitating seamless navigation, and fostering an immersive user experience within our application, resulting in a high-usability application.

### 4.4. Characteristics of the Implemented System

The individual parts of the application can be characterized as follows:

#### 4.4.1. Loading Data

The first step that the users must perform in the application is the aforementioned data retrieval. The users can either choose to use the data already loaded in the application (data from VÚSCH) or to load their own data. If they use the first option, they are allowed to use other functionalities of the application (which we will describe later) immediately. If the users choose to load their own data, they must perform an additional action. This involves defining how the individual numeric attributes that are part of the dataset will be loaded and transformed into categorical ones. The users have two options for such a transformation, which include direct retyping of numeric attributes to categorical attributes, which is useful if the numbers represent a different value, such as a category. The second option is discretization into intervals of equal width or depth. It is necessary to define an adjustment for each numeric attribute separately. In cases where the retrieved dataset does not contain any numeric attributes, the transformation process is not needed. After the data loading is finished, or users chose the preloaded dataset, they can proceed to the sections of the app dedicated to descriptive data analytic models.

#### 4.4.2. Search for Association Rules

After successful data retrieval, the users can choose whichever section is required from the menu on the top of the screen. In the case of the section dedicated to finding association rules, the users have several input elements to use. These are located both in the side menu and the main panel of the user interface, and they are intended for customizing the content of the input dataset. The users are also able to define threshold values for the input model parameters in order to search for association rules such as minimum support, minimum confidence, or left-hand side rule length. They can also restrict the occurrence of selected attributes on the right-hand side of the association rules. It has several outputs that are located in the main part of the user interface.

**Figure 3:**
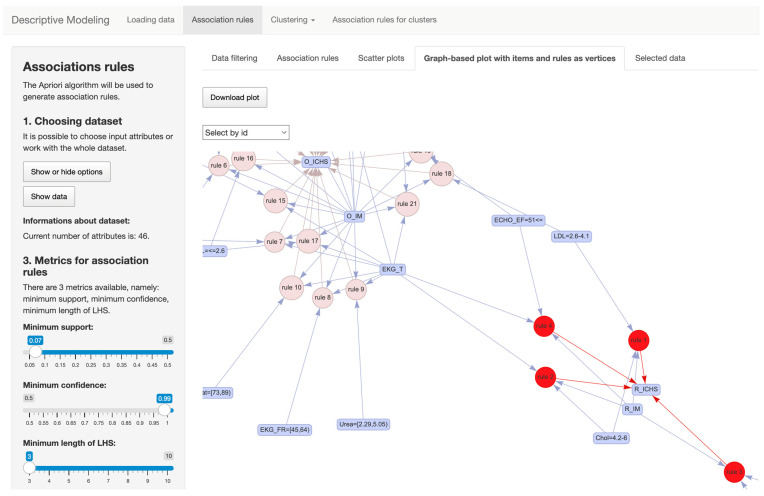
Graph-based plot with items and rules as vertices.

The outputs can include the found association rules, presented in the form of a table, or interactive visualizations via which it is possible to better understand the content of the obtained results. Specifically, we mean the visualization of the relationships between the individual attribute values that occurred in the generated association rules and the association rules themselves, as seen in Figure 3. Additionally there is a dot plot, which is used in this context to show the distribution of the different association rules based on metrics such as support, confidence, and lift, which in different combinations can occur on the x and y axes. When working within the association rules tab, users can choose their own set of attributes from the dataset in the menu, located on the left part of the screen and marked with Number 1, and they also receive information about the number of currently chosen attributes. After that, pressing the button *Show data* transfers them to the main screen and to part Number 2, where users can use filters to select individual records. If they are satisfied with their choice, pressing the button *Confirm!* confirms the choice of records and transfers users to the tab marked *Selected data*. Users can then proceed to settings in the left menu (which is numbered 3), where they set metrics for association rules, e.g., minimum support, minimum confidence, and length of LHS (Left-Hand-Side of association rules). Number 4 contains restrictions of RHS (Right-Hand-Side of association rules), where users can choose which attributes will not appear in the association rules. This feature is implemented based on doctor requests, where they want to filter these rules based on the known factors of cardiovascular diseases. Number 5 in the menu contains the *Render!* button, which renders association rules based on the settings chosen in previous parts.

#### 4.4.3. Application of Clustering Methods

In the implementation of our system, the second option in the top menu facilitates the application of clustering methods to the input data. This functionality is pivotal for organizing and understanding complex datasets by grouping similar records into clusters. To compute the distance between records accurately, we adopted the Gower distance metric [41], which is renowned for its efficacy in scenarios where the input dataset comprises attributes of varying data types. Users are granted the flexibility to customize the input dataset according to their specific requirements or to conduct analyses to determine the optimal number of clusters. This can be achieved through the utilization of well-established methods, such as the Silhouette method or the Elbow method, which aid in identifying the most suitable clustering configuration.

**Figure 4:**
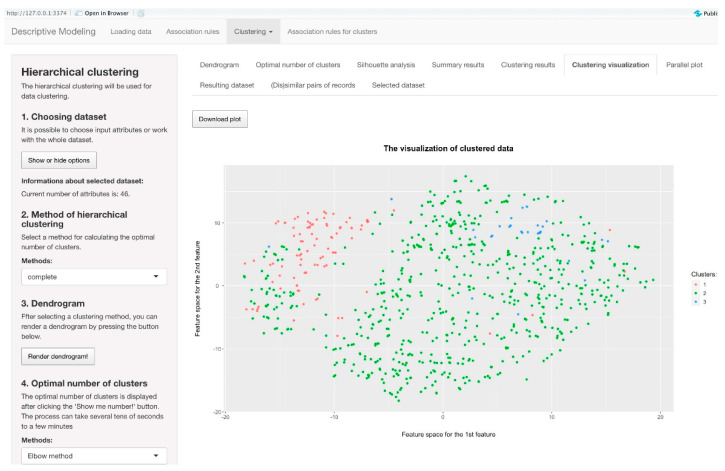
Clustering visualization.

For hierarchical clustering, users are provided with additional customization options, including the selection of a specific method tailored to their analytical needs. Users can choose and filter patient records in part Number 1 in the left menu. They can proceed with Number 2 to choose the method of clustering and Number 3 to render the dendrogram. Number 4 in the left menu calculates the optimal number of clusters with the Elbow method, Silhouette method, and Silhouette analysis. In Number 5, users choose the number of clusters that they want to be generated, confirming it in Number 6 by pressing the *Render!* button. Furthermore, users have the capability to generate a dendrogram visualization, which offers a graphical representation of the hierarchical clustering process, enabling a visual understanding of cluster relationships and structures. This section of the application yields multiple outputs designed to provide comprehensive insights into the clustering results. Among the available outputs are individual validation criteria, which offer quantitative assessments of clustering quality and aid users in evaluating the effectiveness of the clustering algorithm. Additionally, users can access visualizations depicting the distribution of data points across clusters, as seen in Figure 4, facilitating the identification of cluster boundaries and patterns within the data. Furthermore, users gain access to the resulting dataset, which delineates the assignment of each record from the input dataset to specific clusters, thereby providing clarity on which record belongs to which cluster. Finally, users are presented with information regarding the records that are most representative in individual clusters, showing the characteristics of each cluster and identifying records that exhibit the lowest amount of similarities within clusters.

#### 4.4.4. Search for Association Rules within Defined Clusters

In the final segment of our app, the third part is dedicated to the discovery of association rules within the clusters defined in the previous section. Once users have reviewed and are satisfied with the clustering results, they have the option to delve deeper into each individual cluster and uncover meaningful associations among the data points. This functionality is particularly valuable for extracting actionable insights and identifying patterns specific to each cluster, thereby facilitating targeted decision making and tailored interventions.

Similar to the clustering section, users are presented with a familiar interface that allows for seamless interaction with the data and analysis results. The inputs and outputs remain consistent with those defined in the preceding paragraphs, ensuring continuity and ease of use throughout the application, which is important for high usability. However, in this part of the application, users are provided with the flexibility to focus exclusively on association rules found within the clusters of their choice. This approach enables users to refine their analysis and concentrate on the clusters that are most relevant to their specific objectives or areas of interest. As with the clustering section, users can explore a variety of outputs designed to provide comprehensive insights into the association-rule-mining process. These outputs include individual validation criteria, visualizations of rule distributions, detailed summaries of rule characteristics, and information on the most significant associations within each cluster. By offering a comprehensive suite of outputs tailored to the user’s selected clusters, the application helps users to find valuable insights and derive actionable recommendations, ultimately driving informed decision making.

#### 4.4.5. Estimation of the Optimal Number of Clusters

**Figure 5:**
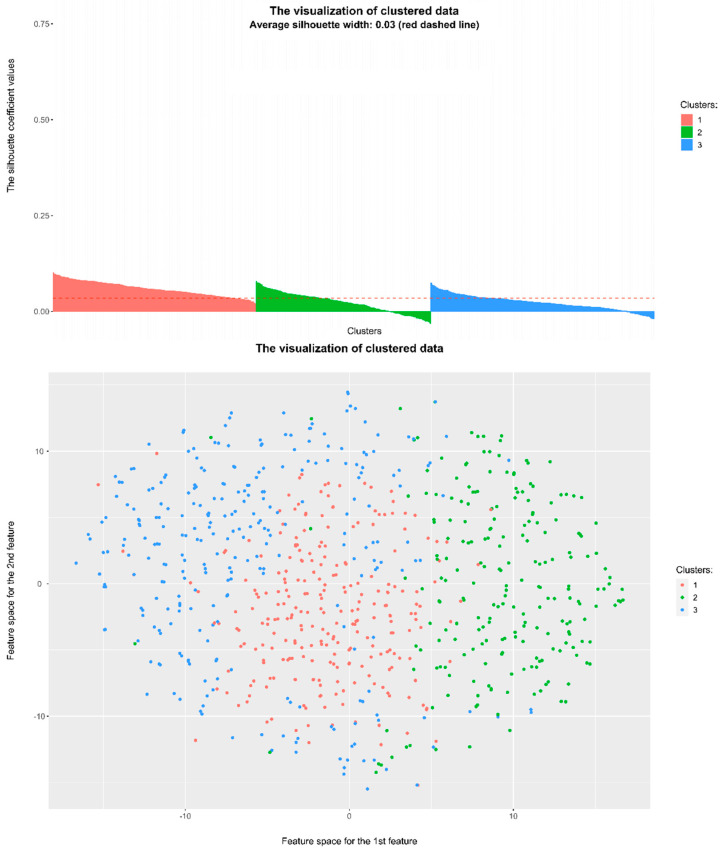
Optimal number of clusters according to the silhouette method.

In the clustering application section, users are provided with the functionality to estimate the optimal number of clusters for their dataset. This critical step aids users in determining the appropriate granularity for partitioning their data and identifying meaningful patterns within the dataset. To facilitate this process, the application offers two popular methods for estimating the optimal number of clusters that we have mentioned before: the Silhouette method and the Elbow method. The Silhouette method evaluates the quality of clustering by assessing how well each data point fits within its assigned cluster relative to other clusters. This method generates silhouette scores for different numbers of clusters, with higher scores indicating better cluster cohesion and separation. By visualizing these scores in the form of bar graphs, users can gain insights into the optimal number of clusters that maximizes overall cluster quality, as seen in Figure 5.

Similarly, the Elbow method provides users with a heuristic approach to determining the optimal number of clusters based on the distortion or within-cluster sum of squares (WCSS) values. This method calculates the WCSS for different numbers of clusters and identifies the point at which adding more clusters no longer significantly reduces the WCSS. The resulting visualization, presented as bar graphs again, enables users to identify the “elbow” point, which suggests the optimal number of clusters.

The system recommends a range of clusters, from two to 10, offering users flexibility in exploring different clustering configurations. However, it is important to note that these recommendations serve as guidance rather than strict rules. Users may choose to further analyze the clustering results or adjust the number of clusters based on domain knowledge or specific project requirements. Ultimately, the goal of providing these recommendations is to assist users in making informed decisions about the optimal clustering configuration for their data, thereby facilitating more effective data analyses and interpretations.

#### 4.4.6. Saving the Obtained Results

An important functionality of our system is the export of obtained results from the application environment, enabling users to utilize and share their findings beyond the application. This feature enhances the versatility, accessibility and usability of the analysis results, empowering users to integrate them into various workflows and presentations. For tabular data, or records displayed within the application, users have the flexibility to export them in widely used formats such as .xlsx or .csv. This capability facilitates easy integration with external tools or further analysis. Additionally, users can selectively export a subset of records, enabling focused analysis or sharing of specific data points of interest.

Graphical visualizations generated within the application can be exported in the .png format, preserving the visual representation of data patterns and insights. This facilitates the incorporation of visualizations into reports, presentations, or publications, ensuring clarity and accessibility of the analysis results. Furthermore, the application features interactive visualizations, allowing users to dynamically explore and interact with data visualizations. To preserve the interactivity of these elements outside the application environment, users can export them in the .html format. This format retains the interactive features of the visualizations, enabling users to continue exploring and analyzing the data even after exporting it. By offering a range of export options tailored to different types of analysis results, the application empowers users to effectively communicate their findings and insights to stakeholders, colleagues, or clients. This seamless integration with external tools and formats enhances the usability and impact of the analysis results, facilitating informed decision making and driving actionable outcomes.

## 5. Application and Usability Testing with Medical Professionals

### 5.1. Application Functionality Testing with Medical Professionals

According to the Nielsen Norman group [42], the best testing results come from testing with at least five users and running as many small tests as can be afforded. We decided to test the app with more than five respondents to receive more feedback. The respondent group that participated in the 1st testing of the implemented application consisted of seven members, all of whom were specifically doctors working in various departments of the Louis Pasteur University Hospital (UNLP) in Košice and in the VÚSCH (East Slovak Institute of Heart and Vascular Diseases), which is also located in Košice. The respondents were of different genders and ages, and the length of their activity in the medical sphere was also different.

The initial testing phase comprised prepared scenarios that involved tasks developed in collaboration with a specialized cardiology department physician. This testing was important, as we needed to thoroughly test all the core app functionalities with professionals. The testing scenario consisted of two tasks and involved the respondent answering by choosing one of the three options offered. The way in which the wording of the tasks was formulated corresponded to the primary target group of the implemented application, which consisted of the aforementioned physicians or medical professionals. It is also important to note that the questionnaire covered all the important parts of the application in its content and, therefore, in the way the tasks were constructed. Given that the respondents were guided, if necessary, in testing the application and thus in completing the given tasks, we can say that they were able to examine the app well and in depth. However, what was relevant was that all respondents went through the prepared tasks and had their own experience of working with all parts of the application. This fulfilled the prerequisites for qualified answers to the questions in the second part of testing the app with non-professional users.

### 5.2. Application Usability Testing with Medical Professionals

In the second phase of testing, our focus shifted towards evaluating the usability of the implemented application through the administration of a standardized questionnaire. To achieve this, we employed a framework consisting of ten predefined questions known as the System Usability Scale (SUS questionnaire) [28]. This approach was selected for its ability to provide valuable insight into the subjective evaluation of the application’s usability from the perspective of the users. This phase of testing is closely intertwined with the previous one, as it allows testers to assess the usability of the application based on the firsthand user experience gained during the execution of the assigned tasks. By soliciting feedback through the SUS questionnaire, we aimed to gather comprehensive insights into various aspects of usability, including ease of use, efficiency, learnability, and overall user satisfaction. This structured approach enabled us to identify strengths and weaknesses in the application’s usability, paving the way for targeted improvements and enhancements. Moreover, by correlating the findings from the usability evaluation with the outcomes of the task-based testing, we were able to gain a holistic understanding of the application’s performance and user experience. This iterative approach towards testing and evaluation ensures that the application not only meets functional requirements but also aligns with user expectations and preferences, ultimately leading to a more user-centric and effective solution. The System Usability Scale (SUS) score is calculated, as we mentioned before, based on users’ responses to a set of ten questions designed to assess the usability of a system. Each question in the SUS questionnaire is scored on a 5-point Likert scale, ranging from Strongly Disagree (1) to Strongly Agree (5). The scoring process involves the following steps:

The SUS yields a single number representing a composite measure of the overall usability of the system being studied. Note that scores for individual items are not meaningful on their own. To calculate the SUS score, first sum the score contributions from each item. Each item’s score contribution will range from 0 to 4. For items 1, 3, 5, 7, and 9, the score contribution is the scale position minus 1. For items 2, 4, 6, 8 and 10, the contribution is 5 minus the scale position. Multiply the sum of the scores by 2.5 to obtain the overall value of SU. SUS scores have a range of 0 to 100.

1. Odd numbered questions: For questions 1, 3, 5, 7, and 9, responses are reverse coded, so the score contribution is the scale position (1–5) minus 1;
2. Even numbered questions: For questions 2, 4, 6, 8 and 10, the contribution is 5 minus the scale position (1–5);
3. Calculating scores: Each individual question is then assigned the score based on whether it is an odd or even numbered question;
4. Summing scores: The scores for all ten questions are summed together to obtain a total score;
5. Adjusting scores: Multiply the sum of the scores by 2.5 to obtain the overall value of usability. SUS scores have a range of 0 to 100.

Therefore, the final SUS score represents an aggregate measure of the perceived usability of the system, with higher scores indicating greater usability. A score above 68 would generally be considered above average, while a score below 68 suggests that improvements are needed to enhance usability.

With an average app rating score of 74.64 from all of seven respondents, our application falls within the range of 68 to 80.3 on the rating scale. According to the grading system, this places our application in the “B” category, indicating a high level of usability. In verbal terms, our software can be described as “good”, further affirming its usability. Additionally, the positive feedback from respondents reinforces the usability of our application. Out of the seven respondents, five have reported finding the implemented system easy to use. This demonstrates a majority agreement on the user-friendly nature of our application. Furthermore, an equal proportion of users fully agree that the functions within the application are very well integrated. This cohesion and seamless integration of features contribute to a positive user experience, further validating the usability of our application. Overall, these findings affirm that our application not only meets usability standards but also provides a user-friendly and integrated experience for its users.

## 6. Extension of Van Welie Layered Model of Usability

**Figure 6:**
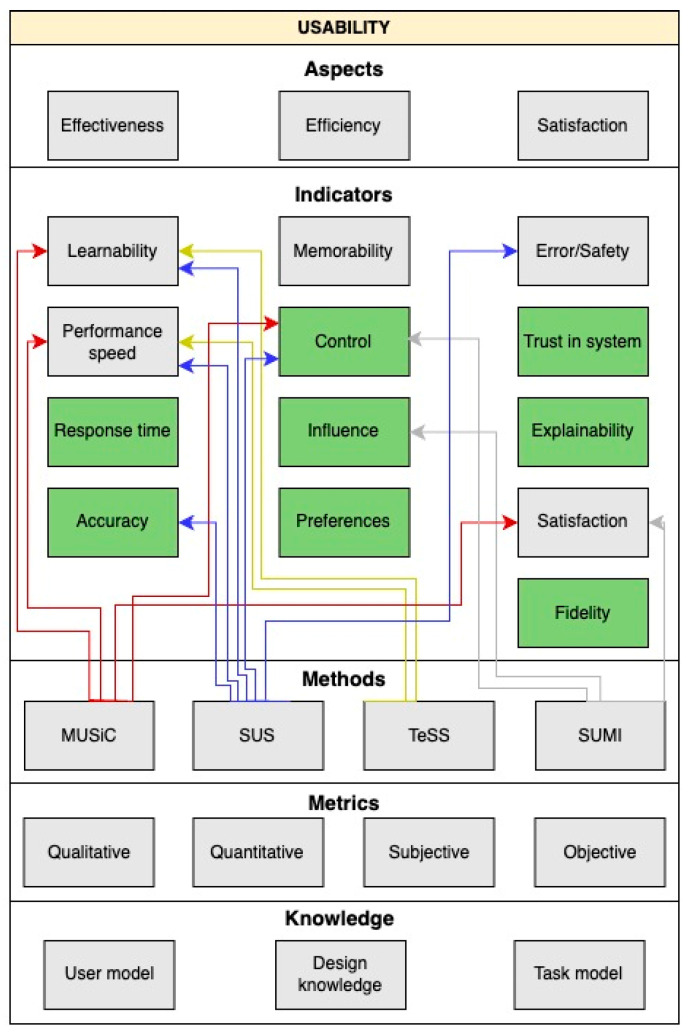
Proposed extension of Van Welie layered model of usability.

After reviewing several studies focused on usability research, its aspects, and specific metrics, we concluded that there is no universal model by which we can measure usability. As usability is a very complex concept, and because it is necessary to consider in which environment, system, and for which users we need to evaluate it, it is necessary to choose appropriate metrics and methods to measure usability. Therefore, in this part of the research, we have summarized current knowledge and defined a new, more universal usability measurement model for data analytics, according to which we can more easily determine which methods are suitable for usability measurement based on our requirements. We will start from Van Welie’s model, mentioned in Section 2, which is a general usability model that we will extend to specific indicators of usability aspects that have their own measurement methods (Figure 6).

Based on the studies examined, we concluded that, based on the study [43], it would be appropriate to add an indicator of “explainability”. The indicators of “control” and “influence” consistently appear in the studies [28,44,45]. The new indicators determining the “accuracy” of the system came from the study [18], while “fidelity” and “response time” are mentioned in the other research article [46]. In the parts highlighted with green in Figure 6, new indicators are shown with which we can better focus on usability measurement in data analytics models. Thanks to these indicators, we will focus more on the explainability of the system, its accuracy, what response it has, and whether it looks trustworthy and preferable to the user or whether its operation is easy for the user. Indicators in Figure 6. are arranged in columns under the aspects, so we know which indicators to focus on if we want to measure the usability of an aspect pertaining to our system.

After grouping the examined methods for usability measurement, we can see that we can measure some indicators by several methods. To measure objective metrics, i.e., to determine the usability of the system from a technical point of view, such as speed, simplicity, error rate or accuracy of the system, it is advisable to use the methods like SUS, MUSiC, or TeSS. Conversely, if we need to measure how the user sees the usability of a given system from a subjective point of view, it is advisable to use the SUMI method, which focuses on satisfaction, ease of operation, and user influence.

The newly developed model shows that the methods we analyze focus primarily on examining aspects such as system efficiency and effectiveness. Methods for measuring user satisfaction with the system did not focus so much on this aspect. A study [43] describing the explainability divides the methods of measuring this indicator into qualitative and quantitative categories. We can also apply these two groups of division of methods to our model, as we have divided the indicators into objective and subjective point of view. Among qualitative methods, we can, by definition, include the SUMI method, as well as the SUS method, depending on what aspect (objective/subjective) of the system the questionnaire is oriented towards. Among quantitative methods, we can, by definition, include the methods SUS, TeSS, and MUSiC.

If we were to divide the chosen measurement methods according to the evaluation of the explainability aspect, the TeSS method would belong to Human Based Evaluation, as the TeSS experiment is carried out with laymen and includes an essential element of learning in addition to the need to obtain specific information. We could include MUSiC, SUMI, and SUS methods in the application-based evaluation, as all three methods are performed only after users have experienced working in a given system. However, the SUS method can also be included in the evaluation based on the functionality of the system, as it can determine how to create a suitable, easy-to-use system based on what users would expect from a functional system.

## 7. Testing Usability of DESSFOCA App

The second and most important part of the testing with which respondents worked is to measure the usability of the DESSFOCA application from the non-professional users’ point of view. This allows us to determine the real usability of the app, which is not biased towards domain expertise. This part is divided into subsections that measure the usability of the application in terms of working with association rules, the application of hierarchical clustering methods, and also the general usability of the application. It is also important to mention that we mapped the proposed extension of the Van Welie model on aspects, which we rigorously tested and determined, if they were really applicable and could improve usability. When evaluating usability, 17 respondents replied. Respondents were able to either choose an answer with an attributable value (“I strongly disagree”—1, “I rather disagree”—2, “I am not sure”—3, “I rather agree”—4, “I completely agree”—5) regarding which best described their view of the question, and they also had the option of an open answer.

Evaluating the questionnaire is straightforward. We calculate the score for usability itself, or a given indicator, as the arithmetic mean of the answers in a given question and in the group of questions pertaining to a given indicator. We convert the resulting value to a percentage score. The result will be the compliance rate of the given usability indicator. In this way, we can also measure the usability of the entire system, as, at the end, we calculate the arithmetic mean of all indicators. In order to be able to express usability verbally, not just numerically, we can also take the evaluation from the SUS questionnaire, as we mentioned in the evaluation with medical professionals. The SUS method considers the type of question and whether it is asked positively or negatively. The questions in our questionnaire are asked only in a positive way, so, in this case, we can apply the calculation of the score by the arithmetic mean of the answer weights. The result of the evaluation of the questionnaire is considered average or good if its score is about 68%. If the result of the questionnaire is above the threshold of 80.3%, the evaluated application is considered above average. Conversely, if the score is less than 51%, the application is considered insufficient and is deemed not usable.

### 7.1. Usability of the Application When Working with Association Rules

In this section, we created five questions to find out how respondents worked with the features that the application offers when working with association rules. The option to choose the type of dataset was useful for the vast majority of respondents, which can be judged by the high score value of 93%. According to the high score of respondents, 86%, the function of filtering records was also useful, as they were able to simplify their work and thus work with fewer records. When asked to limit the right side of the rule, respondents’ answers were more uncertain, as the answers “I am not sure” and “I rather agree” were predominant. This may be because the tasks were simple and only partially covered this feature. This feature is more designed for professionals who use it frequently. The visualizations, as well as the actual understanding of the association rules, were well understood by respondents. The overall usability score for association rules featuresd in the app is 83.8%.

### 7.2. Usability of the Application When Applying the Clustering Methods

For tasks with hierarchical clustering, we created four questions to which respondents gave us their feedback. The most useful feature was the dataset selection feature, which reached a score of 88%. The visualizations of hierarchical clustering were not so well understood by non-professional users, as this feature only reached a value of around 77%. For a better understanding, it would be necessary to describe in more detail what the graph shows and which values are relevant to the user. The graphs can become very crowded and hard to read, even for experts in data analytics, so this part is absolutely understandable. Despite the fact that the evaluation of the success of tasks when working with hierarchical clustering came out better than tasks with association rules, the usability of this part of the app reached a slightly lower score of 80%.

### 7.3. Usability of the DESSFOCA App

For clearer usability measurements, we divided the questions asked into groups depending on which indicator they belonged to. We focused mainly on the new indicators from the proposal of the Van Welie layered model of usability extension (see Section 6).

#### 7.3.1. Ease of Use and Overview in the System

The ease of use for respondents depends mainly on whether the controls and functions are easy to understand. Since respondents were guided by the description of the steps to use the application in order to reach the result, they considered the DESSFOCA app to be relatively simple to use. As soon as respondents in the second part of the tasks (hierarchical clustering application) had to work without assistance, it became more difficult for them to complete the tasks. The tasks to be answered by respondents were designed mainly for non-experts, a group which includes the testers. The response to the apps ease of use would probably be lower if it worked with more demanding features. In contrast, the design of the application and clarity were understandable to respondents, and they considered the application to be consistent. The overall rating of this indicator is 68.46%, which means that it needs improvement in this area. We would recommend adding explanatory notes to help better understand the attributes, or a more detailed description of how to work with the record filter and attribute tagging.

#### 7.3.2. Explainability

When it comes to explainability, we mainly focused on whether the respondent needs a user manual and possibly the help of an expert, as well as whether it is necessary to already have some knowledge to work with the application or if it is self-explanatory. Up to 82.3% of respondents perceived that it is necessary to have instructions for using the application, with the help of a technician/expert being found not to be necessary. The respondents stated that even the initial quick training was sufficient for understanding the basic functions with which they worked. When asked whether it would be necessary to have previously acquired knowledge before using the application, the answers were very diverse; therefore, we assume that it depends on what functions the user will work with. Explainability based on answers is 48.25%, which is a very low value. This indicator was not fulfilled correctly by the application. In practice, we would recommend adding explanations to the application’s functions, as well as illustrative examples of how to work with the application. We also recommend adding explainability features on descriptive models used within the app. We assume that the value of this indicator would be higher if the respondents were experts from practice and not non-expert users.

#### 7.3.3. Accuracy of Results and Time of the Response

After examining the results of the respondents’ responses to this indicator, we found that it had the best rating so far, reaching 82.93%. The response speed is acceptable, and respondents did not experience any problems with generating tables or graphs. According to them, the functions with which the respondents worked are well integrated. The visualization types that were applied to the application were rendered quickly and looked clear. The positive response to this question can also be attributed to the fact that the respondents had what to look for in the graphs explained to them. The application itself does not provide a description of the graphs, which is important for users and should have been implemented. The question asking whether, according to the respondents, the results are accurate had the most neutral answers, as the results were not compared to anything.

#### 7.3.4. SYSTEM Features and Sustainability

The respondents could not assess whether the preset dataset in the DESSFOCA application contains all the necessary attributes, as this question should be asked of an expert in the given domain. As the respondents used the basic functions, they needed to work with the preloaded dataset, and the answers to the question about the use of functions were mostly positive. To improve this function, it would be advisable to insert the possibility of adding a custom attribute according to the user’s needs. The ability to export results in several formats, such as .xlsx or .csv, is very useful, as the user can work with these results and format them outside the application. The application has a well-described procedure of steps in which user should proceed to achieve the result, so the response to the logical sequence was high, reaching 82.35%. The overall rating of this indicator was 82.63%.

#### 7.3.5. Trust in the System

The questions on this indicator were directed mainly at whether the respondents find this application easier and more efficient, whether they would use it in the future, and whether they would recommend it to colleagues/classmates/researchers. Trust in the application had an average value of 68.90%. We assume that this average value is mainly influenced by the fact that the respondents’ frequent answers regarding future use of the application were neutral, as all the respondents, while testing the application, were non-experts and will probably not work in the medical field in the future. We can see from testing with medical experts that, when we conducted the testing with domain experts, the response to the usefulness of the application was much higher. We conclude, based on the answers from our respondents from testing with experts, that the application will significantly simplify the work in comparison to if the evaluation of the results was performed manually. This statement had the highest value in the group focusing on the system trust indicator, reaching 82.35%.

#### 7.3.6. Trust in Result

The trust in result indicator consisted of two questions that focused on whether respondents felt confident using the app. Since they worked only with a predetermined dataset, the trust in the results was high at 88.23%. The answers to the feeling of self-confidence when using the application were different, which we can see in the final value of this question (69.41%). This value was preceded by whether respondents had a good understanding of the procedure and explanation of how to navigate the application. The total value of this indicator was 78.82%.

#### 7.3.7. Influence and Influence on Decision

With this indicator, most of the respondents perceived that the system simplifies and facilitates their work with data, as the application provides several functions for working with data; therefore, this question had a score of 78.82%. Since the respondents will not continue to work with this data and the application, the influence of a user’s decision has an average value of 69.41%. The error rate was higher than we expected for some of the entered tasks, which may also be due to the fact that the application only provided a limited amount of help messages, such as how to proceed and correct the error in the event of it. Of course, we only noticed a reading comprehension problem in a small amount of answers. The value of this indicator reached 72.54% during testing.

#### 7.3.8. Preference and Quality of the Solution

The last indicator we focused on during testing was whether the application provided everything the user expected from it. We have seen comments indicating that, when downloading a chart as an image, the chart was not downloaded correctly or the downloaded document was empty. However, the application met most of the users’ expectations and therefore had a response value of 77.64%. Since the application provided only minimal notifications in cases of bad filtering of records or other functions, we recommend adding error messages to indicate why the step cannot be performed by the application and how the user can fix it. This feature would make the application faster and easier to use. This indicator acquired a value of 72.93%.

#### 7.3.9. Discussion and Results Summary

The “Explainability” indicator had the lowest value. As we already mentioned in regard to the characteristics of this indicator, it is very important to focus on the quality of the explanation, as this indicator also affects the overall impression of the application/system and trust in the system. This statement is also supported by the study [46], where the authors noted that a good explanation should be understandable for people and accurately describe the model behavior in the entire feature space, which increases the value of the fidelity indicator among users. Explainability is very subjective, and the perceived quality of the explanation is contextual and depends on the expertise of users, the explanation itself, as well as the type of information the user is interested in. In practice, we would recommend adding a user manual on how to navigate the application, illustrative examples of how to work with the application, explanations of the application’s functions, as well error messages and help on how to fix them.

The indicators “System functions and sustainability” and “Accuracy of results and part of response” had the highest values, and the percentage difference was minimal. The high value of the first mentioned indicator can be attributed to the fact that the tasks that the respondents had to process mainly covered the basic functions of the system. Therefore, respondents encountering a problem with a lack of functions in working with the application could not happen. Since they were working with a small amount of data, the response of the system and the generation of tables and graphs was acceptable.

The overall usability of the application reached a value of 73.61%. Based on the aforementioned articles and studies, we consider the application to be average, and it received an evaluation as being “good”, with a score close to 68%. Table 1 shows us the values of individual usability indicators. We also evaluated the usability of the system from the point of view of the most basic aspects, such as “Effectiveness”, “Efficiency” and “Satisfaction”, based on the newly created usability model. We found that the indicators “Explainability” and “Fidelity in the result”, which were poorly fulfilled according to the respondents, cause low user satisfaction with the application/system. Conversely, the indicators “Control”, “Influence” and “Preferences”, which belong to the aspect of effectiveness, had the highest values. The indicators “Accuracy” and “Response time” also had well-evaluated values, and therefore we consider this system to be effective. The overview of all usability indicators can be seen in Table 1.

## 8. Conclusions

The use of data analytics in the field of medicine can be considered to be a beneficial and effective approach. The information and knowledge gained has a wide range of applications, whether in the early diagnosis of diseases or their treatment and prevention. We have implemented a new app named DESSFOCA (Decision Support System for Cardiologists). The implemented software consists of three main parts that focus on finding association rules, applying clustering methods, and finding association rules amongst defined clusters. By appropriately interpreting the obtained results, either in textual form or in the form of visualizations, it was possible to verify their relevance from a medical point of view, a task that also involved the participation of a doctor who specialized in cardiology. The software solutions usability was tested with a target user group consisting mainly of doctors or people working in a medical environment. The results obtained can be evaluated as positive, with an average score of 74.64 points, which corresponds to a grade B, which can be interpreted as good usability of the application.

We continued our research with tasks that consisted mainly of examining aspects, indicators, methods, and metrics according to which we could more easily determine which methods are suitable for measuring usability based on our requirements. We discovered that they were based on the well-known Van Welie model, a layered model of usability. We proposed an extension of this model with specific indicators of aspects of usability, which have their own measurement methods. We tested the DESSFOCA app again, now with non-professional users, in order to determine the general usability of the app and to approve the proposed extension of the Van Welie model. We prepared tasks with which the respondents had to work and describe their evaluation based on testing out the system. The evaluation of the system reached 73.61% in its average score of usability; even so, the system is usable, and it has appropriately selected and sufficient functions. During the evaluation, we focused on each newly proposed indicators in our extension of the Van Welie model separately, so that we could find out which indicators need to be improved. At the end of the work, after evaluating the usability of the DESSFOCA, we proposed recommendations for improving both the value of the indicators and also the overall usability, such as adding a manual for users and improving error messages for improvement and acquisition of higher usability value. Additionally, we also conclude that our extension of the Van Welie layered model of usability is sufficient and correct.

## Figures and Tables

**Table 1:** Usability indicators score overview.

Usability Indicators	Value of Usability Indicator
Accuracy of result and response time	82.93%
System functions and sustainability	82.63%
Fidelity (trust) in result	78.82%
Preferences and quality of solution	72.93%
Influence and Influence on decision	72.54%
Trust in system	68.90%
Easy control and easy system overview	68.46%
Explainability	48.25%

## Data Availability

Due to patient confidentiality, it is impossible to provide access to the data used in the development of the app, even though they are anonymized.
